# Disadvantages of publishing biomedical research articles in English for non-native speakers of English

**DOI:** 10.4178/epih/e2015021

**Published:** 2015-05-01

**Authors:** Mohsen Rezaeian

**Affiliations:** Social Medicine Department, Occupational Environmental Research Center, Rafsanjan Medical School, Rafsanjan University of Medical Sciences, Rafsanjan, Iran

**Keywords:** Biomedical research, Medical writing, English language, Disadvantages

## Abstract

**OBJECTIVES:**

English has become the most frequently used language for scientific communication in the biomedical field. Therefore, scholars from all over the world try to publish their findings in English. This trend has a number of advantages, along with several disadvantages.

**METHODS:**

In the current article, the most important disadvantages of publishing biomedical research articles in English for non-native speakers of English are reviewed.

**RESULTS:**

The most important disadvantages of publishing biomedical research articles in English for non-native speakers may include: Overlooking, either unintentionally or even deliberately, the most important local health problems; failure to carry out groundbreaking research due to limited medical research budgets; violating generally accepted codes of publication ethics and committing research misconduct and publications in open-access scam/predatory journals rather than prestigious journals.

**CONCLUSIONS:**

The above mentioned disadvantages could eventually result in academic establishments becoming irresponsible or, even worse, corrupt. In order to avoid this, scientists, scientific organizations, academic institutions, and scientific associations all over the world should design and implement a wider range of collaborative and comprehensive plans.

## INTRODUCTION

English has become the most frequently used language for scientific communication in the biomedical field. Evidence suggests that a constantly increasing trend for English to predominate in biomedical publications may have begun in approximately 1900 [1], and this trend has a number of advantages [2]. For example, publishing in English not only helps to communicate scientific findings more easily, but also it makes scientific findings more widely accessible and therefore more likely to be cited. Furthermore, English is a living language, meaning that it contains prefixes, suffixes, and other word-building elements that enable the logical construction of new words.

These factors explain why scholars in most developed and developing countries where the mother tongue is not English have a tendency to publish their findings in English [3,4]. Moreover, universities in such countries encourage this tendency by preferentially promoting faculty members who have publication records in prestigious English-language journals.

This trend unfortunately also has many disadvantages, which need to be dealt with very carefully and delicately by scientific communities all over the world. Otherwise, the disadvantages of this trend might eventually outweigh its advantages, resulting in the academic establishment becoming irresponsible or, even worse, corrupt, especially in developing countries where the mother tongue is not English. Therefore, in this piece, I will focus on the most important disadvantages of this trend, especially in medical research, and provide some suggestions of how to avoid or mitigate those disadvantages.

## DISADVANTAGES

One of the most important disadvantages of this trend is that scholars in developing countries where English is not the mother tongue might overlook, either unintentionally or even deliberately, the most important local health problems. Such problems might be overlooked either because they are not suitable for publication in prestigious international English-language journals or because the authors fail to convince the editors and/or reviewers of these journals of the importance of such issues. As a result, in countries with a higher burden of some important but neglected local issues such as poverty, malnutrition, and infectious diseases [5-7], or other issues that negatively impact public health, such as natural or man-made disasters [8-11], fewer research projects are carried out in response to these problems.

This dynamic might result in the academic establishment in these countries becoming irresponsible. A previous study has shown that, despite an increasing quantity of publications on health policy and systems research in low-income countries, only 4% of these publications had a first author from the countries in question. Moreover, the capacity for conducting local research has not sufficiently increased in low-income countries [12].

The second most important disadvantage is likewise related to limited budgetary support for medical research. Due to budget shortages, scholars in developing non-English-speaking countries are not able to carry out groundbreaking research, even on issues that are relevant for publication in prestigious international journals. Therefore, they have a low rate of publication within prestigious medical journals [13].

The third most important disadvantage is that academic English writing is very difficult for many non-English-speaking scholars [14]. The most important reason for this is that one cannot directly translate from another language into English. Certain accepted terms exist for a range of concepts and the incorrect use of such terms can alter the meaning, many rules have extensive exceptions that can only be learned through rote memorization, several difficulties exist in tense usage, and various words have different meanings depending on the context. It has been established that research funding and English proficiency are strongly related to publication in the top-ranked general medical journals [15].

Furthermore, a number of non-English-speaking scholars, especially from developing countries, are also unfamiliar with some critical issues in publication ethics. The most important reasons for this are that publication ethics is not usually taught in universities and that few or no governing bodies are in place [16]. Therefore, some non-English-speaking scholars might innocently or deliberately violate generally accepted codes of publication ethics and commit research misconduct, such as failing to disclose all conflicts of interest, committing plagiarism, or, even worse, engaging in salami publication, publishing duplicate publications, or engaging in data fabrication or falsification [17].

These dynamics might result in the academic establishment in these countries becoming corrupt. A study investigating the retraction of publications in MEDLINE from 1966 to 2008 for plagiarism demonstrated that the retraction rate was higher among first authors affiliated with lower-income non-English-speaking countries [18].

There are a number of other fundamental problems which, taken together, create a constellation of disadvantages in the current system of academic publishing. Problematic dynamics that exacerbate this situation include the lack of academic publishing platforms, the lack of up-to-date skills and techniques, the lack of comprehensive public health databases, inadequate information-seeking behavior, an inadequate capacity for teamwork, and other similar problems (Table 1).

## SUGGESTIONS

In my opinion, scientists, scientific organizations and associations, and academic institutions all over the world should think carefully about these disadvantages and try to overcome them through collaborative and comprehensive planning. For example, carrying out collaborative research between English-speaking scholars from developed countries and non-English-speaking scholars from developing countries is a well-established course of action. Although successful examples of such collaboration exist throughout the world [19-22], the current extent of such collaboration is not sufficient.

We should also think of other practical plans. For example, shifting more of the health research budgets of international organizations, such as the World Health Organization or non-governmental organizations, towards relevant health problems within developing countries could be considered. One of the prerequisites for allocating such budgetary funds should be that the work must be carried out collaboratively among English-speaking scholars from developed countries and non-English-speaking scholars from developing countries. Alternatively, international and non-governmental organizations could request non-English-speaking scholars to follow standard publication guidelines throughout the entire course of their research [23].

Moreover, prestigious international English language medical journals should implement a plan to select more editors or associate editors from developing countries in order to reflect their responsibility towards the health of all the people of the world [24]. In a sense, we are all living in a global village. Therefore, such journals should also consider including a section focusing on the publication, either in print or online, of well-conducted research that highlights relevant local and national health problems in the developing world.

Prestigious international English-language medical journals, especially those published by eminent publishers, should also adopt a new peer review policy in which it is not possible to reject a manuscript only because of weaknesses in academic English [25]. Instead, they should provide affordable English-language editing opportunities for worthwhile manuscripts that are submitted by non-English-speaking scholars. International organizations could potentially shift some of their research funding towards this important endeavor [26].

Similarly, a parallel policy should be adopted by the prestigious open-access medical journals. Moreover, they should discount or even waive their publication charges for well-researched manuscripts. Although such policies have been already put in place by some journals,which waive publication charges for authors in genuine financial hardship [27], such policies are not universal.

It is absolutely necessary to remember that most of the research which is carried out in non-English-speaking countries might receive dramatically less funding. For example, more than half of my research proposals have received less than US$1,000 of funding. Therefore, it is simply impossible for scholars from these countries to pay up to US$580 [28] for English-language editing and/or up to US$2,900 [29] for publication charges.

In addition, scientists, scientific organizations, academic institutions, and scientific associations from all over the world, such as the World Association of Medical Editors, should also develop and implement a collaborative plan to detect open-access scam/predatory journals in the biomedical domain and publicize them, especially for non-English-speaking scholars [30].

Last but not least, some other practical suggestions would include conducting English-language writing courses for biomedical scholars all over the world, urging them to consult books and articles on English academic writing [31], and asking them to make use of self-employed science editors. Biomedical research organizations and universities, especially in non-English-speaking developing countries, could also employ experienced English editors [32].

The implementation of the above suggestions, along with investments in biomedical research infrastructure [33,34] and developing appropriate courses in research methodologies, may help overcome other problems that have been mentioned, such as the lack of academic publishing platforms, the lack of up-to-date skills and techniques, the lack of comprehensive public health databases, inadequate information-seeking behavior, an inadequate capacity for teamwork, the inadequate appreciation of diverse types of research misconduct, and other problematic dynamics (Table 1).

## CONCLUSION

Despite the advantages associated with non-English-speaking scholars publishing scientific findings in English, we should be aware of its disadvantages as well. These disadvantages could eventually result in academic establishments becoming irresponsible or, even worse, corrupt, especially in developing countries where the mother tongue is not English. In order to avoid this, scientists, scientific organizations, academic institutions, and scientific associations all over the world should design and implement a wider range of collaborative and comprehensive plans.

## Figures and Tables

**Table 1.** Summary table of problems and suggestions associated with the current system of English-language academic publication

Problems	Suggestions
Limited medical research budgets	Allocating adequate budgetary resources for medical research and carrying out collaborative research
Overlooking the most important local health problems	Allocating adequate health research budgets and carrying out collaborative research
Low record of publicationin prestigious medical journals	Allocating adequate budgetary resources for medical research, carrying out collaborative research, and selecting more scholars from developing countries to serve as the editors and associate editors of prestigious health journals
Unfamiliarity with academic English writing	Implementing English-language writing courses, using self-employed science editors, and providing affordable English-language editing services
Unfamiliarity with some critical issues in publication ethics	Urging scholars to use relevant publication guidelines, implementing appropriate research methodologies courses, and carrying out collaborative research
Publications in open-access scam/predatory journals	Detecting and publicizing open-access scam/predatory journals, and waiving publication charges in prestigious open-access journals
Lack of academic publishing platforms, lack of up-to-date skills and techniques, lack of comprehensive public health databases, inadequate information-seeking behavior, inadequate capacity for teamwork	Investment in biomedical research infrastructure, allocating adequate budgetary resources for medical research, carrying out collaborative research, and implementing appropriate research methodologies courses
